# Topological characterisation and identification of critical domains within glucosyltransferase IV (GtrIV) of *Shigella flexneri*

**DOI:** 10.1186/1471-2091-12-67

**Published:** 2011-12-22

**Authors:** Anesh Nair, Haralambos Korres, Naresh K Verma

**Affiliations:** 1 Division of Biomedical Science and Biochemistry, Research School of Biology, Australian National University, Canberra ACT 0200, Australia

## Abstract

**Background:**

The three bacteriophage genes *gtrA, gtrB *and *gtr_(type) _*are responsible for O-antigen glucosylation in *Shigella flexneri*. Both *gtrA *and *gtrB *have been demonstrated to be highly conserved and interchangeable among serotypes while *gtr_(type) _*was found to be specific to each serotype, leading to the hypothesis that the Gtr_(type) _proteins are responsible for attaching glucosyl groups to the O-antigen in a site- and serotype- specific manner. Based on the confirmed topologies of GtrI, GtrII and GtrV, such interaction and attachment of the glucosyl groups to the O-antigen has been postulated to occur in the periplasm.

**Results:**

In this study, the topology of GtrIV was experimentally determined by creating different fusions between GtrIV and a dual-reporter protein, PhoA/LacZ. This study shows that GtrIV consists of 8 transmembrane helices, 2 large periplasmic loops, 2 small cytoplasmic N- and C- terminal ends and a re-entrant loop that occurs between transmembrane helices III and IV. Though this topology differs from that of GtrI, GtrII, GtrV and GtrX, it is very similar to that of GtrIc. Furthermore, both the N-terminal periplasmic and the C-terminal periplasmic loops are important for GtrIV function as shown via a series of loop deletion experiments and the creation of chimeric proteins between GtrIV and its closest structural homologue, GtrIc.

**Conclusion:**

The current study provides the basis for elucidating the structure and mechanism of action of this important O-antigen modifying glucosyltransferase.

## Background

Shigellosis, or bacillary dysentery, is caused by members of the genus *Shigella*. This genus belongs to the Gram-negative bacterial family *Enterobacteriaceae*, and is divided into 4 species: *S. flexneri, S. dysenteriae, S. boydii*, and *S. sonnei*, all of which cause shigellosis. *Shigella flexneri*, the species responsible for the highest mortality rate, is endemic in most developing countries. There are 15 known *S. flexneri *serotypes, which differ in their virulence, prevalence, distribution and O-antigens [1,2]. The O-antigen is the distal capping moiety of the bacterial

lipopolysaccharide (LPS), a molecule that extends from the bacterial surface. The O-antigen backbone consists of repeating units of the tetrasaccharide *N*-acetylglucosamine-rhamnose I-rhamnose II-rhamnose III [3]. This basic backbone, serotype Y, is present in all serotypes except serotype 6 and 6a. The addition of glucosyl and/or O-acetyl groups to different sugars in the tetrasaccharide unit by one of several linkages gives rise to different serotypes. Glucosylation can occur in any one of the four residues that are present in the tetrasaccharide unit. O-acetylation was thought to occur only on the rhamnose III residue, resulting in the group 6 epitope [4]. However, recent studies have revealed that a degree of O-acetylation may be occurring on the other sugars as well [5,6].

The three genes involved in O-antigen glucosylation are *gtrA, gtrB*, and *gtr_(type)_*. They are encoded by temperate bacteriophages and located downstream of the *attP *site in the bacteriophage genome [2]. While *gtrA *and *gtrB*, which encode for GtrA and GtrB, are highly conserved and interchangeable among serotypes [7-10], *gtr_(type) _*, which encodes for Gtr_(type) _protein, is serotype-specific and is unique to each bacteriophage. It is hypothesised that GtrB catalyses the transfer of glucose from UDP-glucose to bactoprenol phosphate to form UndP-β-glucose in the cytoplasm [10]. This molecule is then flipped by GtrA into the periplasm before the glucosyl residue is attached by the Gtr_(type) _to the growing O-antigen unit [11]. This attachment is thought to take place in the periplasm. GtrIV adds a glucosyl residue to *N*-acetylglucosamine of the O-antigen repeat unit via an a1,6 linkage, thus converting serotype Y to serotype 4a.

In this study, a dual reporter system consisting of alkaline phosphatase (*phoA*) which is in-frame with the β-galactosidase a-fragment (*lacZα*) developed by Alexeyev and Winkler [12] was used to determine the membrane topology of GtrIV. Alkaline phosphatase (AP) is an *E. coli *enzyme that is only active when it is localised in the periplasm as the mature part of PhoA is oxidised such that the cysteine residues are able to form disulfide bridges. This enables PhoA to fold correctly [12]. In contrast to AP, β-galactosidase (BG) is only active in the cytoplasm as the a-fragment must interact with the cytoplasmically expressed w-fragment of the enzyme to cause α-complementation [12].

Here we report that GtrIV clearly differs from the topologies of the other Gtrs (GtrI, GtrII, GtrV, GtrX) but is strikingly similar to the confirmed topology of GtrIc, a newly discovered glucosyltransferase, which has 11 transmembrane regions, a short cytoplsmic C-terminal tail and two large periplasmic loops [13]. Based on the fact that glucosylation is thought to occur in the periplasm [10], we also show that the two large periplasmic loops of GtrIV are important for its stable assembly in the membrane and function by creating GtrIV proteins that have either the N-terminal periplasmic loop or various segments of the C-terminal periplasmic loop deleted, resulting in the abolishment of O-antigen modification from serotype Y to serotype 4a.

## Methods

### Growth conditions of bacterial strains

All bacterial cultures were grown aerobically at 37°C on either liquid Luria-Bertani (LB) agar plates or in liquid LB medium. Antibiotics such as chloramphenicol (Sigma), kanamycin (Sigma) and ampicillin (Sigma) were added to solid and liquid media when required. The final concentrations of these antibiotics were 25 mg/mL, 50 mg/mL, and 100 mg/mL, respectively. Bacterial plates were stored for up to 3 weeks at 4°C in the cold room. The *E. coli *strains used in this study are derivatives of *E. coli *K-12. XL1-Blue MRF' *E. coli *was used for routine cloning procedures while JM109 *E. coli *was used for cloning of topology constructs to be screened on Dual indicator (DI) plates that contain the chromogenic substrates X-phos (5-bromo-4-chloroindol-3-inolyl phosphate disodium salt, Sigma) for alkaline phosphatase and Red-Gal (6-chloroindol-3-inolyl-β-D-galactoside, Research Organics) for β-galactosidase as outlined by Alexeyev and Winkler [12].

### DNA cloning techniques

All plasmids constructed in this study were derived from the cloning vector pBC SK+ (Stratagene). They were maintained in JM109 cells and isolated by means of using either the QIAGEN MiniPrep Kit or the alkaline lysis method adapted by Sambrook and Russell [14] from Birnboim and Doly [15]. Restriction enzymes were purchased from Fermentas or New England Biolabs (NEB). Amplification of specific genes was performed via PCR by using *Pfu *ultraII poymerase (Stratagene) and specific oligonucleotide primers purchased from Sigma-Aldrich (Additional file 1, Table S1). T4 DNA ligase (Promega) was used for all ligation reactions as specified by Promega. Electrocompetent cells for bacterial transformations were prepared according to Dower *et al*. [16] and transformations were carried out as described by Sambrook and Russell [14] and Dower *et al*. [16]. DNA sequencing was performed at the Biomolecular Resources Facility, John Curtin School of Medical Research, The Australian National University. Point mutations within *gtrIV *were introduced using the QuikChange Site-Directed Mutagenesis Kit (Stratagene). The resulting amplicon was then treated with *Dpn*I before being introduced into XL1-Blue MRF' *E. coli *that repairs nicks. The mutant constructs were sequenced to confirm the desired mutation.

### Computer analysis of protein topology

Six topology prediction programs that were available online were used to examine the GtrIV protein sequence for the presence of hydrophobic regions. They were HMMTOP [http://www.enzim.hu/hmmtop/] [17], SOSUI [http://bp.nuap.nagoya-u.ac.jp/sosui/] [18], TMpred [http://www.ch.embnet.org/software/TMPRED_form.html] [19], DAS [http://www.sbc.su.se/~miklos/DAS/] [20], TopPredII [http://bioweb.pasteur.fr/seqanal/interfaces/toppred.html] [21] and finally, TMHMM [http://www.cbs.dtu.dk/services/TMHMM/] [22].

### Creating a topology template and generation of constructs for topology studies

Before exonuclease III (Exo III) deletion and PCR-based fusions can be carried out to create *gtrIV*-*phoA/lacZ *truncations, an appropriate template had to be created such that *gtrIV *is in tandem with the *phoA/lacZ *dual reporter. pNV1090, constructed by Korres and Verma [23], contains *gtrV *in tandem with *phoA/lacZ*. The majority of pNV1090, with the exception of *gtrV*, was amplified with forward primer 1090*Xba*IFnew containing an *Xba*I site that anneals directly upstream of *phoA*/*lacZ *in pNV1090 and reverse primer 1090*Nhe*IRnew containing an*Nhe*I site that anneals upstream of *gtrV*. Similarly, *gtrIV *was amplified from pNV739 (containing *gtrIV*) using a forward primer gtrIVNheIFnew (5'-TCAGCTAGCCTCGGTGGTGTGCAGCTC-3') and a reverse primer gtrIVXbaIRnew (5'-TCATCTAGACCCCCCAGGATAACTGTGGG-3'). Ligation of both amplicons created pNV1473 (Additional file 2, Figure S1) which contains a *Pst*I site closest to *phoA/lacZ *that leaves an Exo III-resistant 3' overhang and a *Bam*HI site closest to *gtrIV *that provides an Exo III-susceptible 5' overhang. Upon creation of pNV1473, the plasmid was linearised by a *Pst*I/*Bam*HI double digestion and progressively deleted from the end of *gtrIV *using the Erase-a-base kit from Promega. PCR was also used to create *gtrVI-phoA*/*lacZ *fusions. In this approach, the majority of pNV1473 was amplified using the forward primer phoF (5'- GTTCTGGAAAACCGGGCTGCTCAG-3'), which anneals at the beginning of the *phoA*/*lacZ *sequence, and a reverse primer that anneals at the point of interest in *gtrIV*.

In order to make sandwich fusions, *Nru*I sites were first incorporated into the *gtrIV *sequence in pNV1473 via site-directed mutagenesis. *Nru*I digest screening was used to identify successfully mutated constructs and they were tested for functionality by slide agglutination (as described below). *phoA*/*lacZ *was excised from pMA632 by either *Sma*I/*Eco*RV, *Ehe*I/*Ecl*136II or *Stu*I/*Nru*I double digests, such that *phoA*/*lacZ *fragment would be in-frame when ligated with the *Nru*I digested fragments. JM109 was transformed with the ligation mixtures and plated onto DI plates. Colonies that displayed colouration were investigated further using restriction digests and then verified by sequencing using M13R or the PHOSEQNewR primers.

### Quantifying alkaline phosphatase and β-galactosidase activities

Alkaline phosphatase (AP) and b-galactosidase (BG) assays were carried out to quantify the AP and BG activities, respectively. Both assays were performed in parallel with duplicates for each experiment. After obtaining the AP and BG activities for each fusion in the data set, the normalised activity ratios (NAR) were calculated as follows [24,25]:

NAR = (Alkaline phosphatase activity/highest Alkaline phosphatase activity)/(b-galactosidase activity/highest b-galactosidase activity)

### Functional analysis of GtrIV

The function of Gtr proteins were tested by transformation into SFL1616, a serotype Y *Shigella *strain containing chromosomally-encoded *gtrA *and *gtrB*. Serotype conversion (from serotype Y to serotype 4a) indicated a functional GtrIV protein. Slide agglutination assays were carried out by bacteria grown to log phase at 37°C (A_600 _0.8-1.0), mixing the culture gently on a glass slide with Type IV antisera (Denka Seiken) and observing for agglutination [26].

### Bacterial lipopolysaccharide (LPS) preparation

This method was adapted from Hitchcock and Brown [27] with several modifications. Overnight cultures were diluted 1/100 in LB containing the appropriate antibiotics and incubated for 2 - 3 h at 37°C until OD_600 _of 0.6 was reached. 1.5 ml of culture was then spun down and the pellet was resuspended in 80 μl sample loading buffer (4% SDS, 160 mM Tris-HCl, 20% glycerol, 10% β-mercaptoethanol). Proteinase K was then added to each sample at a concentration of 50 mg/ml and the samples were incubated overnight at 56°C and stored at -20°C. Before samples are run on 12% SDS-PAGE gel, 0.5 μl β-mercaptoethanol was added to the samples and boiled for 10 min.

### Membrane protein preparation

The membrane protein isolation method is a modified version of that described by Morona *et al*. [28]. Cells were grown in LB to mid-log (OD_600 _of 0.6) and pelleted using a Sorvall SLA1500 rotor (7,000 rpm, 10 min, 4°C) and resuspended in 1 ml of 20% (w/v) sucrose, 30 mM Tris-HCl pH 8.1, transferred to SS-34 tubes and chilled on ice. 0.1 ml of 1 mg/ml lysozyme in 0.1 M EDTA pH 7.3 was added to the cells for 30 min on ice. The cells were collected again as described above using a Sorvall SS-34 rotor and the pellet frozen for 30 min at -80°C. The pellet was thawed and resuspended vigorously in 6 ml 3 mM EDTA, pH 7.3. The cells were completely lysed by passing them twice through a French Press at 15,000 psi. Unlysed cells and inclusion bodies were removed by slow centrifugation as described above (7,000 rpm, 10 min, 4°C). Membrane proteins were sedimented by high speed centrifugation using a 50Ti or 80Ti rotor spun at 35, 000 rpm for 90 min at 4°C. The pellet was then resuspended in 200 μl sterile Milli Q H2O.

### Western blotting of proteins

Western blotting was carried out as described by Thanweer *et al *[29]. For membrane preparations 10 μl of each 2 mg/ml sample was loaded and for LPS samples, 10 μl of each LPS extraction was loaded. Primary antibodies used were either Mouse anti-alkaline phosphatase (Chemicon) diluted 1:1000 for membrane protein preparations, Type IV antisera (Denka Seiken) diluted 1:100 or serotype Ic-specific MASFIc monoclonal antibody (Reagensia) diluted 1:500 for bacterial LPS extractions. The secondary antibodies used were Goat anti-mouse IgG horse radish peroxidase (HRP)-conjugated (Sigma) diluted 1:8000 for membrane protein preparations while Goat anti-rabbit HRP-conjugated Ig (Sigma) diluted 1:1000 and anti-mouse IgM peroxidise conjugate (Sigma) diluted 1:5000 were used for profiling bacterial LPS extraction. The reactions were detected either by X-ray film (GE Healthcare) or viewed under the Fusion Chemiluminescence Camera (Fisher Biotech).

## Results and discussion

### Determination of GtrIV topology

A predicted topology model for GtrIV was created based on the results given by the various web-based prediction programs (Additional file 1, Table S2). There was no common prediction amongst the programs. HMMTOP and TMPred both predicted that GtrIV has 8 transmembrane helices. TopPred and TMHMM predicted 9 transmembrane helices while SOSUI and DAS predicted 10 transmembrane helices and 11 transmembrane helices, respectively. Of all the programs, HMMTOP and TMHMM both predicted that the N-terminus was cytoplasmic. TopPred predicted that the N-terminus was in the periplasm while the rest of the programs did not provide any information about the localisation of the N-terminus. From the information that has been gathered from the confirmed topologies of GtrI, GtrII and GtrV, a large periplasmic C-terminal tail was expected. By collating the data from all the programs, a consensus model was created. In this model, GtrIV is shown to have 10 transmembrane helices, a cytoplasmic N-terminus, a small cytoplasmic C-terminal region, and two large periplasmic loops between transmembrane helices I and II, and transmembrane helices VII and VIII (Additional file 3, Figure S2**)**.

To experimentally verify this hypothetical GtrIV model, three different fusion approaches were used. The nested deletion system, which utilised exonuclease III (Exo III) deletion from the end of the *gtrIV *gene followed by fusion with *phoA/lacZ*, resulted in about 200 coloured colonies on DI plates. Subsequent screening of these colonies and sequence analysis yielded 19 unique in-frame GtrIV/PhoA-LacZ fusion proteins. The colourations displayed by these 19 fusions were in agreement with the topology model. A blue colouration, which is caused by the breakdown of X-phos by PhoA, indicated a periplasmic localisation. Likewise, LacZ breakdown of Red-Gal yielded a red colouration, indicative of a cytoplasmic localisation. A purple colouration, on the other hand, indicated that the fusion was within the transmembrane helix as a combination of both red and blue colouration is present, thus giving the colony its purple appearance. To further check if these fusions are in agreement with the model, alkaline phosphatase activity (AP) and β-galactosidase (BG) activity for these 19 in-frame fusions were measured via enzyme assays for AP and BG activity and the Normalised Activity Ratios (NARs) were calculated. Table 1 shows the NAR values. The NAR values were generally consistent with the previously observed coloured colonies. Interestingly, only one in-frame red fusion was obtained by Exo III deletion. Most of the Exo III mediated fusions were seen to occur in the two large putative periplasmic loops. Given that the consensus model shows that all the cytoplasmic loops are relatively small, there will only be a smaller chance of an in-frame fusion occurring within these loops. According to Alexeyev and Winkler [30], NARs greater than 2:1 or lower than 1:2 indicate that at least 67% of the reporter activity is properly localised. Alexeyev and Winkler [12] also stated that the reliability of NARs in providing data about reporter membrane localization is related to the size and diversity of the set of fusions and can be tested by choosing the second highest reference point in calculating the NARs. This was performed (results not shown), and the NARs remained consistent with the previously determined localization of the reporter thus fortifying our confidence in the finalized topology of GtrIV. All the computer programs did not give the same results and therefore, this highlights the importance of verifying the computer based consensus model. Fusion I200 reported a red DI colouration and a NAR of 1:34. Although this is evidence of cytoplasmic localisation, it showed a high AP activity of 22. Seeing as it also had a substantially high BG activity of 132, it suggests that this fusion is in agreement with the topology model and located in close proximity to the cytoplasmic face of transmembrane helix IV. Alexeyev and Winkler [12], state that purple fusions result as an association of the fusion to a transmembrane region. This is not an exclusive rule, as seen with fusion I200. The red DI colouration could be a result of the fusion's overwhelmingly higher BG activity coming to the fore. A total of 4 purple fusions were obtained from Exo III deletion. Fusions A15, Q186, V359 and Q393 have NAR values of 2:1, 1:11, 5:1 and 1:4, respectively. Fusion V359 shows periplasmic localisation while fusions Q186 and Q393 show cytoplasmic localisation. Though their NAR values suggest that these fusions are either localised in the periplasm or cytoplasm, it can be seen in Table 1 that among these four fusions, the lowest average AP activity is 9 while the lowest average BG activity is 8. This therefore indicates that some level AP and BG activity is present in these fusions. The varying NAR values obtained is attributed to the fact that the %AP values are obtained using highest AP activity of 976, while the %BG values are obtained using the highest BG activity of 155.

**Table 1:** Analysis of *gtrIV-phoA/lacZ *fusions and *gtrIV-phoA/lacZ-gtrIV *sandwich fusions for GtrIV topology determination

Sample ID	AA^1^	Colour^2^	Average AP^3^	Average BG^4^	%AP^5^	%BG^6^	NAR^7^ (AP:BG)	Location on Model^8^
**Random C-terminal Fusions**								
B1925	A15	Purple	311 ± 9	25 ± 2	32.6%	16.1%	2:1	t.1
B1910	N29	Blue	356 ± 47	1^#^	37.3%	0.6%	52:1	p.2
B1924	P38	Blue	357 ± 44	2^#^	37.5%	1.3%	24:1	p.2
B1907	N41	Blue	296 ± 47	1^#^	31.1%	0.6%	38:1	p.2
B1935	G42	Blue	195 ± 28	3 ± 1	20.5%	1.3%	20:1	p.2
B1906	L56	Blue	133 ± 29	4 ± 1	14.0%	2.6%	5:1	p.2
B1931	F60	Blue	383 ± 25	1^#^	40.2%	0.6%	57:1	p.2
B1912	Q186	Purple	9 ± 91	15 ± 2	0.9%	9.7%	1:11	t.4
B1919	I200	Red	22 ± 4	132 ± 6	2.3%	85.1%	1:34	t.4
B1913	N235	Blue	292 ± 51	0^#^	30.6%	0.0%	> 100:1	p.6
B1926	L240	Blue	394 ± 20	1^#^	41.4%	0.7%	58:1	p.6
B1904	L246	Blue	660 ± 39	1^#^	69.3%	0.7%	97:1	p.6
B1921	N251	Blue	962* ± 59	1^#^	100.9%	0.9%	> 100:1	p.6
B1922	N255	Blue	553 ± 30	0^#^	58.1%	0.1%	> 100:1	p.6
B1908	D261	Blue	385 ± 55	2^#^	40.4%	1.2%	33:1	p.6
B1905	S300	Blue	426 ± 21	4 ± 1	43.6%	2.8%	16:1	p.6
B1923	M310	Blue	589 ± 15	1 ± 1	61.8%	0.1%	> 100:1	p.6
B1920	V359	Purple	269 ± 34	9 ± 1	28.2%	5.5%	5:1	t.6
B1911	Q393	Purple	12 ± 3	8 ± 2	1.2%	4.9%	1:4	t.7
**PCR-Constructed Fusions**								
B2216	V102	Red	2^#^	121 ± 17	0.2%	78.2%	1: > 100	c.3
B1937	D146	Blue	953 ± 35	7 ± 1	100.0%	4.3%	23:1	re (p.4)
B2217	V155	Red	6 ± 2	71 ± 9	0.6%	45.8%	1:76	re (c.4)
B2405	K162	Red	5^#^	11^#^	0.5%	7.1%	1:14	re (c.4)
B2225	D169	Red	3^#^	79 ± 17	0.3%	51.1%	1: > 100	re (c.4)
B1936	D406	Blue	177 ± 40	1^#^	18.6%	0.9%	20:1	p.8
B1914	K437	Red	5 ± 1	155** ± 8	0.5%	100.0%	1: > 100	c.9
**Sandwich Fusions**								
B2187	R93	Red	0^#^	10 ± 2	0.1%	21.3%	1: > 100	c.3
B2190	K117	Red	0^#^	14 ± 2	0.3%	29.4%	1: > 100	c.3
B2143	A181	Blue	135^+ ^± 25	0^#^	100.0%	0.3%	> 100:1	re (p.4)
B2192	R375	Red	1 ± 1	47^++ ^± 7	0.4%	100.0%	1: > 100	c.7

^1 ^Position of the last residue of GtrIV followed by *phoA/lacZ*, ^2 ^Colour of colony as seen on dual indicator plates, ^3,4 ^Activities of the fusions, average of four independent experiments with standard deviations of each activity, ^5,6 ^Percentages of AP and BG activities measured relative to the maximum activity in the set, ^7^Normalised AP:BG activity ratio (NAR) rounded to the nearest integer. ^8^Location of the fusion on the adjusted topological model of GtrIV(Figure 1); c, cytoplasm; p, periplasm; t, transmembrane helix; re, re-entrant loop. *Highest truncation AP, **Highest truncation BG, ^+^Highest Sandwich fusion AP, ^++^Highest sandwich fusion BG. Since the random C-terminal fusions and PCR mediated fusions brought about truncated protein fused to the dual reporter, they were taken as one data set and the highest respective AP and BG values between them was used to calculate the NAR values. The NAR values for the sandwich fusions were calculated separately based on the highest AP and BG values obtained from all the sandwich fusions alone. ^#^Standard deviations were less than 0.5 and not included.

The topology of GtrIV was further elucidated by using two more strategies. These include a PCR-based approach and the use of sandwich fusions. Using the PCR-based fusion technique, it allowed us to exactly fuse the dual reporter to predetermined points in the protein. The second method involved the construction of sandwich fusions. A sandwich fusion can indicate a more accurate representation of topology since the whole protein is present with the dual reporter sandwiched in the middle. These two methods generated a host of constructs. Fusions D146, D406 and K438 were obtained via PCR based fusions. R93, K117, A181 and R375 were generated using *gtrIV/phoA-lacZ/gtrIV *sandwich fusions. A NAR value of 1: > 100 for the PCR-constructed red fusion K438 confirms that the C-terminal end is cytoplasmic. Fusion D406 with a NAR value of 20:1 indicated that loop No. 10 is in the periplasm. While both these fusions satisfied the consensus model, D146 was observed to display blue colouration with a NAR value of 21:1. This fusion was in contradiction to the hypothetical model, which predicts D146 to be localised in the cytoplasm between transmebrane helices IV and V. Sandwich fusion K117 displayed a NAR value of 1: > 100 that corresponded with its red colouration and confirms its localisation to the cytoplasm.

**Figure 1:**
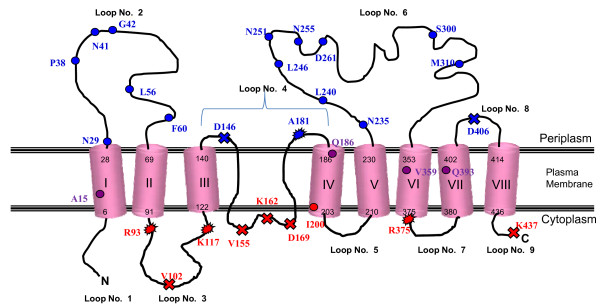
**The final GtrIV topology model**. Final GtrIV topology model after the creation of *gtrIV-phoA*/*lacZ *fusions by the Exo III deletion and PCR-based approaches, and *gtrIV-phoA*/*lacZ-gtrIV *sandwich fusions. In this model, GtrIV is shown to have 8 transmembrane helices and two large periplasmic loops. All Exo III mediated fusions are indicated with the closed circles while PCR mediated fusions are indicated with the crosses. All sandwich fusions are indicated with the stars. Blue coloured stars, circles and crosses depict fusions that have high PhoA NARs and appear blue on DI plates. Similarly, the ones in red depict fusions that have high LacZ NARs and appear red on DI plates. All purple fusions are seen to have occurred within transmembrane helices. The proposed re-entrant loop which traverses the plasma membrane from the periplasm into the cytoplasm, forming a cytoplasmic loop, occurs between transmembrane helices III and IV. All fusions have been labelled with the residue at which the fusion with PhoA/LacZ takes place or, in the case of sandwich fusions, where the dual reporter protein has been inserted.

TMpred and HMMTOP topology programs predicted similar models in which K117 was predicted to be in the cytoplasm and D146 was predicted to be in the periplasm. Truncation fusion V102 which reported a red colouration on DI plates and a high BG NAR compared to AP, strongly suggests that GtrIV had a cytoplasmic loop between transmembrane helices II and III. This fusion was in between both sandwich fusions R93 and K117 that displayed NAR values and colouration favouring a higher BG ratio to AP and a cytoplasmic localisation. According to these results, the model was modified accordingly such that transmembrane helix III from the consensus mode (Additional file 3, Figure S2) was pushed into the cytoplasm along with periplasmic loop No. 4. This consequently would invert transmembrane helix IV, thus allowing the consensus loop No. 5 (containing D146) to be localised in the periplasm. However, inverting loop No. 5 into the periplasm also meant that a large periplasmic loop between the modified transmembrane helices III and IV spanning 47 amino acids was created by including transmembrane helix V of the consensus model. To support this theory, truncation fusions V155 and D169 were then designed so that they were evenly spaced between D146 and A181, to verify the newly modelled periplasmic loop. Both fusions displayed red colouration and NAR values of high BG to AP ratios. In order to satisfy the current results, a re-entrant loop was introduced in the proposed model that completely traverses the plasma membrane into the cytoplasm and loops back up into the periplasm such that V155 and D169 are both localised in the cytoplasm. Analysis of the TMS prediction algorithms indicated that throughout the protein sequence, the transmemrane helices were predicted as clear hydrophobic peaks. However, between amino acid regions P140-I187, two double peaks exist which can be deduced as transmembrane regions (Additional file 4, Figure S3). This trend was also witnessed for the topology prediction of GtrIc [13]. The finalised membrane topology of GtrIV is shown in Figure 1. The blue fusions obtained via Exo III deletions confirmed the periplasmic locations of loop No. 2 and loop No. 6, while blue truncation fusion D406 confirmed that loop No. 8 is in the periplasm. The presence of two large periplasmic loops gives weight to the hypothesis that glucosylation takes place in the periplasm. Therefore, these two loops provide excellent candidates for GtrIV functional studies. Red fusions I200 and K438 confirm the cytoplasmic localization of loop No. 5 and the C-terminus of GtrI, respectively. Sandwich fusion R375 confirms the cytoplasmic loop No. 7, while sandwich fusions R93 and K117 along with truncation fusion V102 show that loop No. 3 of GtrIV is indeed a large cytoplasmic loop, uncharacteristic to the other Gtrs. Such a large cytoplasmic loop is known to exist in another protein involved in *S. flexneri *O-antigen acetylation, O-acetyltransferase (Oac) [29]. Since glucosylation of the O-antigen is thought to occur in the periplasm, it may play a part in helping to maintain the structural integrity of GtrIV during the glucosylation process. Blue fusions D146 and A181, together with red fusions V155 and D169 suggest the presence of a re-entrant loop between the amino acid regions P140 - I187 that starts from the periplasm and traverses through the plasma membrane into the cytoplasm, forming an intramembrane dipping segment which then traverses back into the periplasm. This unique re-entrant loop structure was put forward following comparisons with GtrIc, the closest structural homologue of GtrIV and is hypothesised to provide structural flexibility in order to facilitate interaction between periplasmic loop No. 2 and periplasmic loop No. 6. As mentioned before, GtrIc has an almost identical topology to that of GtrIV. Ramiscal *et al *[13] have also shown that a Gtr-conserved re-entrant loop exists at the lateral centre of GtrIc, between transmembrane helices IV and V. Unlike the re-entrant loops seen in GtrII and GtrV [23,31], Ramiscal *et al *[13] proposed that it formed a double intramembrane dipping region which can be defined as a protein segment that does not completely penetrate the bilayer but briefly enters and exits the same membrane face [32]. In light of the current results and considering that GtrIc is the closest homologue to GtrIV, an intramembrane dipping region seems to exist in GtrIV between residues V155 and D169. As this region is rich in isoleucines (hydrophobic residue), it further supports the presence of such an intramembrane dipping region. However, the presence of a KKE tract within this region could also point to a cytoplasmic loop. Therefore, to reinforce this, a final truncation fusion was designed at K162. This fusion reported a red DI colouration and NAR of 1:14. The relatively low NAR value and BG activity of K162 (11) suggests that while in the cytoplasm, this loop might be folded such that it is close to the plasma membrane. There is a possibility of the presence of an intramembrane dipping region occurring within V155 and D169. In this case, the reported red colouration and subsequent BG enzymatic function may have resulted from the effect of PhoA/LacZ weighing down the region such that it is pulled back into the cytoplasm. This could account for its low BG activity as compared to V155 and D169. However, without the use of visual technology such as X-ray crystallography, such structures are difficult to prove. Hence, we propose the presence of a re-entrant loop between transmembrane helices III and IV of GtrIV, which starts at the periplasmic face of the protein and traverses down into the cytoplasm before making its way back to the periplasm. Re-entrant loops have been documented in eukaryotic glutamate transporters [33], in the bacterial potassium channel KcsA [34] and in various other channels as reviewed by Harris-Warrick [35]. This proposed re-entrant loop may be of minimal influence on catalytic potential but may instead provide GtrIV with the conformational flexibility required to transfer a glucosyl group from a membrane lipid to the O-antigen, as proposed by Korres and Verma [23] for the GtrV protein.

### Identifying regions critical for GtrIV function

**Figure 2:**
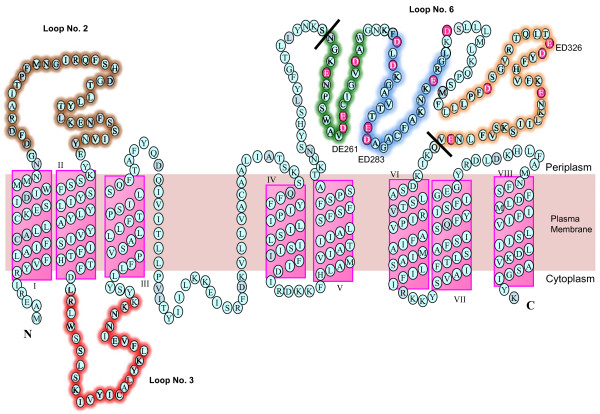
**Model of GtrIV with all residues shown**. Residues in pink are the 15 negatively charged amino acids present in loop No. 6. There are three negatively charged amino acid pairs DE261, ED283 and ED326. Deletion experiments for loop No. 2 involved deleting all the residues highlighted in brown, while a deletion in loop No. 3 was brought about by deleting all the residues highlighted in red. In loop No. 6, 4 different loop deletions were created. To investigate the importance of the negatively charged residues, all residues between the two black lines, which include all 15 negatively charged amino acids were deleted. To further investigate which sets of negatively charged residues were important, amino acids highlighted in green (loop No. 6 partial deletion 3), blue (loop No. 6 partial deletion 2) and orange (loop No. 6 partial deletion 1) were deleted separately.

By identifying critical regions of a protein, it helps provide an insight and may also uncover vital clues to help elucidate its mechanism of action. Residues that are critical within these regions may not only be involved directly in catalysis but also in interacting with substrates and other proteins in a complex. These residues may also be responsible for maintaining the structural integrity of the protein in assisting other critical residues to form the catalytic site [30]. Multiple alignments of both the large periplasmic loops of GtrIV were carried out against the other Gtrs. Although it was found that there was a low level of homology between the periplasmic loops, with the exception of some acidic residues in periplasmic loop No. 2, GtrIV loop No. 2 shares structural homology with the loop No. 2 of other Gtrs [13,23,31,36]. Therefore, it is proposed that periplasmic loop No. 2 in all Gtrs may perform the conserved function of interacting with the UndP-Glucose and possibly recycling the lipid carrier back to the cytoplasmic face of the membrane. To prove the importance of GtrIV loop No. 2 in such function, it was deleted via PCR by using specific forward and reverse primers as outlined in Table S1 (Figure 2). The template DNA used for this deletion PCR was fusion K438 (C-terminal fused to PhoA/LacZ dual reporter protein) obtained during topology studies. This construct was chosen since it can be detected on a Western blot using anti-alkaline phosphatase antibodies to determine expression and assembly in the membrane. Though loop No. 2 is topologically homologous to all Gtrs, GtrIV also has a unique cytoplasmic loop No. 3 consisting of 30 amino acids. Considering that glucosylation occurs in the periplasm, the presence of this large cytoplasmic loop is unusual as it is not seen in the other Gtrs. This drew our attention and we proceeded to create a loop No. 3 deletion to investigate its role in the function of GtrIV (Figure 2). This deletion mutant was created via PCR using specific reverse and forward primers (Additional file 1, Table S1) by utilising the same template that used in creating the loop No. 2 deletion.

**Figure 3:**
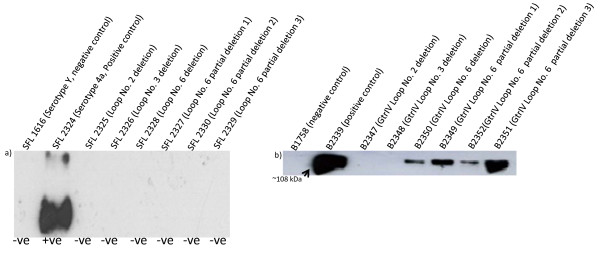
**Functional analysis of deletion constructs**. a) LPS Western blots and slide agglutination results. Deletion constructs were transformed into a serotype Y strain (SFL1616) to test for modification of O-antigen to serotype 4a. SFL 2324 is GtrIV fused to PhoA/LacZ and then transformed into SFL1616. The bright band is indicative of the serotype conversion from serotype Y to serotype 4a. The rest of the deletions show absence of modification to serotype 4a when probed with Type IV antisera. Results of the slide agglutination assays are indicated below the Western blots. A score of +ve indicates a positive result while a score of -ve indicates a negative result. b) Western blots (using anti-alkaline phosphatase primary antibody) performed on the membrane protein extractions of the various GtrIV loop deletion constructs that were fused to alkaline phosphatase. B2347 and B2348 were not detected, indicating GtrIV Loop No. 2 and Loop No. 3 deletions have a direct effect on the assembly of the protein in the cellular membrane. B2349, B2350 and B2352 were detected in lower amounts, the amount of protein that is assembled in the cellular membrane in these three deletion mutants is much lower as compared to the fully intact protein (B2339). The intense band seen for B2351 is almost the same as the fully intact protein.

Loop No. 6 of GtrIV was found to contain 8 aspartic acid residues and 7 glutamic acid residues (15 negatively charged acidic residues in total) spread throughout the loop (Figure 2), and is hypothesised to be responsible for adding a glucosyl residue to the *N*-acetylglucosamine of the O-antigen repeat unit via an α1,6 linkage, thus converting serotype Y to serotype 4a. Korres and Verma [36] have suggested that the acidic residue D380 in GtrV is important for GtrV function because it is believed to interact with the O-antigen to stabilise it so that the glucosyl residue can come in contact with the correct rhamnose. Based on this hypothesis, any one or a combination of the 15 acidic residues may perform this stabilising function in GtrIV. It is also distinctly possible that these acidic residues compensate for the loss of the other acidic residues which would explain why there are three sets of paired acidic residues flanked on either side by a different acidic reside situated 7-9 residues away. To investigate this, a series of loop No. 6 deletions that targeted each set of paired acidic residues together with the flanking acidic residues were constructed (Figure 2). Specific forward and reverse primers were used to amplify the majority of the plasmid, which contains GtrIV which already has PhoA/LacZ fused to its C-terminal (pNV1532) except for the loop segments to be deleted. Positive clones were screened by *Sma*I digest and confirmed by sequencing. A total of 4 clones were constructed with various deletions in loop No. 6. Loop No. 6 deletion, loop No. 6 partial deletions 1, 2 and 3 (Figure 2) were obtained and transformed into SFL1616 (serotype Y) for functionality testing using Type IV antisera. The results of all deletions were negative in a slide agglutination test using Type IV antisera (Figure 3a). Although slide agglutination tests are accurate, there is a possibility that the deletions have caused a decrease in function of the protein. In such a scenario, the agglutination effect would be quite difficult to visualise. To overcome this effect, Western immunoblots using Type IV antisera were performed on bacterial LPS extracted from the deletion mutants to confirm the slide agglutination results (Figure 3a). The results of the LPS immunoblots also showed that all the deletions were unable to convert serotype Y to serotype 4a. Deletion of such large segments of protein may result in protein misfolding. This would in turn abolish GtrIV function. As GtrIV is transmembrane protein, such misfolding would result in reduced protein assembly in the bacterial membrane. To investigate this, we then carried out Western immunoblots on the membrane protein extracts of the various loop deletion constructs to determine whether the deletions had any effect on protein assembly in the membrane using anti-alkaline phosphatise antibodies (Figure 3b). If the deletions had an effect on protein folding, then the misfolded GtrIV deletion mutant might not be assembled on the membrane. Subsequently, this would affect its ability to modify the O-antigen. The Western blot results show that GtrIV was not localised in the cellular membrane when loop No. 2 or loop No. 3 was deleted indicating that these loops are essential for protein structure and assembly. The results also showed that the deletion of loop No. 6 of GtrIV and two of the three partial deletions reduced the amount of protein that was assembled in the bacterial membrane as compared to the fully intact protein. In B2351, the amount of GtrIV that was present in the cellular membrane was almost as high as the amount of the intact GtrIV. This, coupled with the loss of functionality of the deleted protein, provides us with an insight that the missing 19 amino acid segment of loop No. 6 partial deletion 3 could contain residues that may be part of a catalytic site present in the large periplasmic loop No. 6.

**Figure 4:**
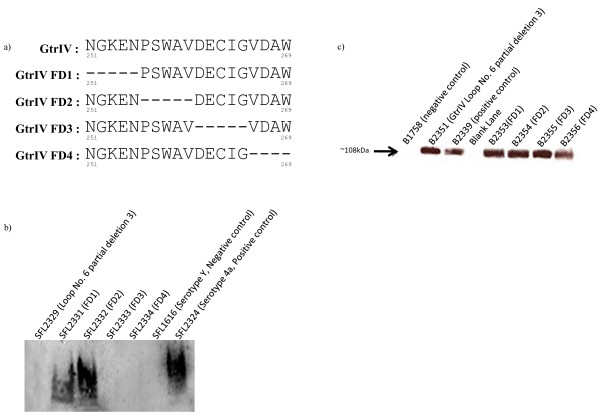
**Further deletions of GtrIV loop No. 6**. a) The sequence of the 19 amino acids located in GtrIV loop No. 6, which were targeted for further deletion. The dashes in the sequences of GtrIV FD1, FD2, FD3 and FD4 indicate the amino acids that were deleted in each deletion set. Amino acid numbers corresponding to the start and end of the peptide sequence are given below their respective residue. b) LPS Western blots of GtrIV deletion constructs that were transformed into SFL1616 (Serotype Y). Deletion constructs FD1(SFL2331) and FD2 (SFL2332) were able to convert serotype Y to serotype 4a, thus indicating functionality. The absence of bands for FD3 and FD4 indicates loss of function for these deletion mutants. c) Western blots of GtrIV Loop No. 6 further deletion constructs. All the further deletion constructs were detected when the membrane extracts were probed with anti-alkaline phosphatase. This indicated that the deletion proteins (which are fused to PhoA/LacZ) are expressed.

Four new deletion constructs that specifically targeted the 19 amino acid spanning region were created (Figure 4a). The strategy used to create these new deletion constructs was identical to the one used in creating the previous loop No. 6 deletion constructs. Resulting clones were screened via *Sma*I digests and confirmed by sequencing. A total of 4 constructs (FD1, 2, 3 and 4) were obtained and used to transform SFL1616. Functional analysis of GtrIV deletion constructs through slide agglutination and LPS Western blots revealed that the introduced deletions FD1 and FD2 were tolerated whereas the deletions present in FD3 and FD4 knocked out GtrIV function (Figure 4b). Western blots of the membrane proteins extracted from these constructs show that all the four further deletion proteins were localised in the membrane (Figure 4c). This is in agreement with the hypothesis that the missing segments may be part of a catalytic site present in the large periplasmic loop No. 6 that might be responsible for interaction with the glucosyl residue to be attached to the O-antigen, but also with potential interactions between GtrIV, GtrA and GtrB

### Investigating periplasmic loop function of GtrIV by using chimeric proteins

As previous studies on other Gtr_(type) _proteins have shown that a degree functional similarity exists between the Gtr_(type) _family, we sought to investigate the role of loop No. 2 of GtrIV by creating chimeric proteins with its closest structural homologue GtrIc. The accepted dogma is that topologically similar N-terminal periplasmic loops in all Gtr_(type) _proteins may perform the conserved functions of interacting with the UndP-glucose and possibly recycling it to the cytoplasmic face. Thus, if both the N-terminal periplasmic loops No. 2 of GtrIV and GtrIc play the same role in O-antigen modification, then swapping these two loops with each other should not abolish function of the native proteins. Chimeric proteins were created via a PCR-based approach, by swapping the N-terminal periplasmic loops of GtrIV and GtrIc to produce chimeric constructs (Table 2). The templates chosen for both GtrIV and GtrIc contained the PhoA/LacZ dual reporter fused to the C-terminal of each protein. Functional analysis of these chimeric proteins were carried out via slide agglutination and LPS Western blotting using Type IV antisera and MASF Ic as the primary antibodies to detect serotype conversion to serotype 4a and serotype Ic, respectively **(**Table 2). The hybrid GtrIc construct containing loop No. 2 of GtrIc failed to convert serotype Y to serotype 4a. Similarly, the hybrid GtrIc construct containing loop No. 2 of GtrIV was unable to convert serotype 1a to serotype 1c. This indicates that although loop No. 2 is topologically conserved, its low sequence identity cannot necessarily establish a functional protein as observed in the GtrV-GtrX Loop No. 2-GtrV chimera [36]. In retrospect, it is possible that the loop No. 2 of both proteins may contain unique amino acid residues that are critical for its conserved interaction with the UndP-Glucose precursor, owing to the fact that the active sites for both these proteins might require slightly different tertiary structures in order to interact specifically with the O-antigen.

**Table 2:** Predicted structures of the four chimeric proteins created in this study between GtrIc and GtrIV

*Chimera Constructs*	*Slide Agglutination*		*Western blots*		
			
			LPS		Membrane
	MASF Ic	Type IV	MASF Ic	Type IV	Anti-PhoA
	+ve	-ve	+ve	-ve	+ve

	-ve	+ve	-ve	+ve	+ve

	-ve	-ve	-ve	-ve	-ve

	-ve	-ve	-ve	-ve	+ve

	-ve	-ve	-ve	-ve	+ve

	-ve	-ve	-ve	-ve	+ve

Points of fusion: a) GtrIc-GtrIV loop No.2-GtrIc chimera:GtrIc-F31/GtrIV-N27, GtrIV-Y68/GtrIc-V100, b) GtrIV-GtrIc loop No.2-GtrIV chimera: GtrIV-M26/GtrIc-H32, GtrIc-N99/GtrIV-S71, c) GtrIc-GtrIV loop No.6-GtrIc chimera: GtrIc-P382/GtrIV-T231, GtrIV-K351/GtrIc-V495, d) GtrIV-GtrIc loop No.10-GtrIV chimera: GtrIV-S229/GtrIc-T231, GtrIc-V381/GtrIV-D352. To test for GtrIc function, the chimera constructs were transformed in to SFL1416 (Serotype 1a) to test for serotype conversion to serotype 1c using MASF Ic. As for GtrIV function, conversion to serotype 4a was tested by using Type IV antisera against constructs that were transformed into SFL1616 (serotype Y). All membrane protein westerns were carried using anti-alkaline phosphatse antibodies.

In parallel to investigating the role of GtrIV loop No. 2, we also investigated the role of the C-terminal periplasmic loop No. 6 of GtrIV. Gtrs I, II, V and × have long periplasmic C-terminal ends that have been hypothesised to be responsible for conferring serotype specificity. In contrast, as both GtrIV and GtrIc have short cytoplasmic C-terminal ends, their specific function is thought to be performed by the large periplasmic loops No. 6 and No. 10 for GtrIV and GtrIc, respectively. By using the same PCR-based approach for the creation of the loop No. 2 chimeras, loop No. 6 of GtrIV was replaced by loop No. 10 of GtrIc and vice versa (Table 2). It was postulated that the hybrid GtrIV containing loop No. 10 of GtrIc would be able to convert serotype 1a to serotype 1c while the GtrIc-GtrIV loop No. 6-GtrIc chimera would be able to convert serotype Y to serotype 4a. Slide agglutination and LPS Western blots revealed absence of modification to respective serotypes. As both large periplasmic loops contain over 100 amino acids each (123 amino acids in loop No. 6 of GtrIV and 120 amino acids in loop No. 10 of GtrIc), each loop can possibly accommodate its own unique tertiary structure that would contribute to the serotype specificity of O-antigen modification. Therefore, in absence of this, GtrIV and GtrIc would not be able to carry out their specific functions. Alternatively, the structural integrity of each chimeric protein may have been compromised with the addition of the foreign loop. To confirm for assembly in the membrane, Western blots (using anti-alkaline phosphatase antibodies) were carried out on the membrane protein extracts of each chimeric protein (Table 2). Of the four chimeric proteins created, only GtrIc-GtrIV loop No. 2-GtrIc chimera was not detected by the blot. Its non-functional hybrid GtrIV counterpart however, was shown to have been assembled in the membrane strongly implying that specific interactions between residues present in its native loop No. 2 are required to form a catalytic site that facilitates O-antigen modification. The absence of assembly in the bacterial membrane of the GtrIc-GtrIV loop No. 2-GtrIc chimera suggests that GtrIV loop No. 2 has compromised the structural integrity of the GtrIc protein. Similarly, loss of function for GtrIc-GtrIV loop No. 6-GtrIc and GtrIV-GtrIc loop No. 10-GtrIV chimeras can be attributed to the importance of the two C-terminal periplasmic loops in conferring serotype specificity through the addition of glucosyl residues to the O-antigen in a site and linkage specific manner. As both loops span more than 100 amino acids each, this would facilitate intra-loop and inter-loop interactions that may contribute to serotype specificity and O-antigen modification.

The present study provides experimental evidence that GtrIV shares structural similarities with GtrIc, thus differing from the rest of the Gtrs. Further studies using site directed mutagenesis of amino acids between D260 to W269 and use of structure definition techniques should allow us to identify critical residues and further define the overall structure which will provide us with critical information to better understand its mechanism of action and catalytic site.

## Conclusion

The structural similarity between GtrIV and GtrIc has been shown in this study by confirming the presence of a periplasmic loop No. 2, a large periplasmic loop No. 6 and a short cytoplasmic C-terminal end in GtrIV. The existence of a re-entrant loop, similar to that seen in GtrIc has also been observed. Two periplasmic regions were identified that could be involved in the attachment of the glucosyl group to the O-antigen. The non-critical nature of the conserved acidic residues coupled by the structural difference of GtrIV compared to the other Gtrs, except GtrIc, could indicate that its mechanism of action may be different from the rest of the Gtrs and may be similar to that of GtrIc. By sequentially deleting loop segments in loop No. 6, the presence of a potential catalytic site located between residues D260 to W269 was hypothesised. To further investigate the roles of conserved or specific functions of the two periplasmic loops of GtrIV, loop swap experiments between the N-terminal periplasmic loops and the C-terminal periplasmic loops were undertaken. The resulting hybrids lost their native function and were unable to substitute function to the other protein. This signifies the importance of both loops in GtrIV function. Furthermore, the identification of critical residues in these regions and further structural studies will provide the basis for the localization of the active site and the elucidation into the mechanism of action of GtrIV.

## Abbreviations

Gtr: Glucosyltransferase; NAR: Normalised activity ratio; AP: alkaline phosphatase; BG: β-galactosidase; LPS: lipopolysaccharide; Exo III: exonuclease III; FD: further deletion

## Authors' contributions

AN contributed to the experimental design of the study, carried out all experiments, analysed results and wrote the manuscript. HK contributed to the experimental design of the study, analysed results and critically revised the manuscript. NKV conceived and directed the study, contributed to the experimental design of the study, analysed results and critically revised the manuscript. All authors have read and approved the final manuscript.

## Supplementary Material

Additional file 1 **Tables S1 and S2**. List of Primers used and Results from the computer programs used to predict GtrIV topology and the basis for each prediction. Click here for file

Additional file 2 **Figure S1**. The structure of pNV1473 depicting *gtrIV *and *phoA/lacZ *in tandem. Click here for file

Additional file 3 **Figure S2**. The consensus topology of GtrIV. Click here for file

Additional file 4 **Figure S3**. Representative computer-based topology predictions of GtrIV. Click here for file
