# Supplementary material for: Topological characterisation and identification of critical domains within glucosyltransferase IV (GtrIV) of Shigella flexneri
Source: BMC Biochem. 2011 Dec 22;12:67. doi: 10.1186/1471-2091-12-67 (PMC3259042; doi:10.1186/1471-2091-12-67)
Supplement: Additional file 1 — Tables S1 and S2. List of Primers used and Results from the computer programs used to predict GtrIV topology and the basis for each prediction. [file 1471-2091-12-67-S1.DOC]

**Table S1:** A list of Primers used in this study

| ***Primer Name*** | ***Primer Sequence (5'-3')*** | ***Annealing site*** | ***Restriction site*** |
| --- | --- | --- | --- |
| *Cloning* | | | |
| *gtrIVNheIFnew* | *TCAGCTAGCCTCGGTGGTGTGCAGCTC* | *Upstream of gtrIVgene* | *Nhe*I |
| *gtrIVXbaIRnew* | *TCATCTAGACCCCCCAGGATAACTGTGGG* | *Downstream of gtrIVgene* | *Xba*I |
| *phoF* | *GTTCTGGAAAACCGGGCTGCTCAG* | *Beginning of phoA/lacZcoding sequence* | *-* |
| *PCR-based Fusions for Topology* | | | |
| *V102HpaIRev* | *TCAGTTAACCACGATTTTGGAAAGAGATGAC* | *In gtrIV gene* | *Hpa*I |
| *Lp5Q146Rev* | *CTGATAAAATGCTGTGAATTG* | *In gtrIV gene* | *-* |
| *V155HpaIRev* | *TCAGTTAACCGGAAGTAAAGTGATTACTATTTG* | *In gtrIV gene* | *Hpa*I |
| *D169HpaIRev* | *TCAGTTAACATCAAACCTTGATATTTCTTTTTTAAG* | *In gtrIV gene* | *Hpa*I |
| *Lp10D407Rev* | *GTCTAAATCTCTATATCCTTC* | *In gtrIV gene* | *-* |
| *GtrIVCtermHpaIR* | *TCAGTTAACCTTATAAATTCCTGATGCTACC* | *In gtrIV gene* | *Hpa*I |
| *Sandwich fusion primers* | | | |
| *GtrIVR93NruIF* | *CACATTTGATCTT****TCGCGA****TGGTCATCTCTTTCC* | *In gtrIV gene* | *Nru*I |
| *GtrIVR93NruIR* | *GGAAAGAGATGACCA****TCGCGA****AAGATCAAATGTG* | *In gtrIV gene* | *Nru*I |
| *GtrIVK117NruIF* | *GTAGAAATAAACAA****TCGCGA****ATATTCTTATCTTTTATTTC* | *In gtrIV gene* | *Nru*I |
| *GtrIVK117NruIR* | *GAAATAAAAGATAAGAATAT****TCGCGA****TTGTTTATTTCTAC* | *In gtrIV gene* | *Nru*I |
| *GtrIVI136NruIF* | *GCTATTTACTCCT****TCGCGA****CTTTCACAATC* | *In gtrIV gene* | *Nru*I |
| *GtrIVI136NruIR* | *GAATTGTGAAAG****TCGCGA****AGGAGTAAATAGC* | *In gtrIV gene* | *Nru*I |
| *GtrIVA181NruIF* | *GCGCAGCGCTAA****TCGCGA****CATCAAAATCTC* | *In gtrIV gene* | *Nru*I |
| *GtrIVA181NruIR* | *GAGATTTTGATG****TCGCGA****TTAGCGCTGCGC* | *In gtrIV gene* | *Nru*I |
| *GtrIVR375NruIF* | *CACTGATTTTTAT****TCGCGA****AAAATATTCTGTTG* | *In gtrIV gene* | *Nru*I |
| *GtrIVR375NruIR* | *CAACAGAATATTTT****TCGCGA****ATAAAAATCAGTG* | *In gtrIV gene* | *Nru*I |
| *GtrIV Loop Deletions* | | | |
| *IVLp2DelSmaIF* | *TCACCCGGGTATAAAAGCTCATTCAGTTATATTC* | *In gtrIV gene* | *Sma*I |
| *IVLp2DelSmaIR* | *TCACCCGGGATTATTCATCATCCATATGTC* | *In gtrIV gene* | *Sma*I |
| *Lp8DelSmaIF* | *TCACCCGGGCAAAAAATAAAAGACTCGC* | *In gtrIV gene* | *Sma*I |
| *Lp8DelSmaIR* | *TCACCCGGGATTGCTTTTATTGTATAGC* | *In gtrIV gene* | *Sma*I |
| *Lp8P_Del1SmaR* | *TCACCCGGGAGGAACATGGAAGGTTGCTTTAG* | *In gtrIV gene* | *Sma*I |
| *Lp8P_Del2SmaIF* | *TCACCCGGGCTTTTGATGCTAAAGCAACC* | *In gtrIV gene* | *Sma*I |
| *Lp8P_Del2SmaIR* | *TCACCCGGGTTTATTTCCCCATGCATCAAC* | *In gtrIV gene* | *Sma*I |
| *Lp8P_Del3SmaIF* | *TCACCCGGGTTTGATTTAGATAAGGGAGC* | *In gtrIV gene* | *Sma*I |
| *IVLp6P.Del3FD1F* | *TCACCCGGGGCTTTTATTGTATAGCAAAGTC* | *In gtrIV gene* | *Sma*I |
| *IVLp6P.Del3FD2F* | *TCACCCGGGGACGAATGCATTGGTGTTGATG* | *In gtrIV gene* | *Sma*I |
| *IVLp6P.Del3FD2R* | *TCACCCGGGATTTTCTTTTCCATTGCTTTTATTG* | *In gtrIV gene* | *Sma*I |
| *IVLp6P.Del3FD3F* | *TCACCCGGGGTTGATGCATGGGGAAATAAATTTG* | *In gtrIV gene* | *Sma*I |
| *IVLp6P.Del3FD3R* | *TCACCCGGGCACGGCCCACGATGGATTTTC* | *In gtrIV gene* | *Sma*I |
| *IVLp6P.Del3FD4R* | *TCACCCGGGACCAATGCATTCGTCCACGGC* | *In gtrIV gene* | *Sma*I |
| *Chimera Studies* | | | |
| *GtrIVLp2BglIIF* | *TCAAGATCTAATGGCGACTTCGATAGGGC* | *In gtrIV gene* | *Bgl*II |
| *GtrIVLp2MluIR* | *TCAACGCGTATACTCATAGTTCACAATTGAAC* | *In gtrIV gene* | *Mlu*I |
| *GtrIVMluIF* | *TCAACGCGTTCATTGAGTTATATTCTTTATTTATACG* | *In gtrIV gene* | *Mlu*I |
| *GtrIVBglIIR* | *TCAAGATCTCATCCATATGTAAATTGACTCC* | *In gtrIV gene* | *Bgl*II |
| *GtrIcLp2BglIIF* | *TCAAGATCTCACCATGGAAGCTGGTCAGG* | *In gtrIc gene* | *Bgl*II |
| *GtrIcLp2MluIR* | *TCAACGCGTATTGAAATCGTGACTTATATAAAATATAG* | *In gtrIc gene* | *Mlu*I |
| *GtrIcBodyMluIF* | *TCAACGCGTGTTACCGTTAAGTTATTAGCG* | *In gtrIc gene* | *Mlu*I |
| *GtrIcVectorBglIIR* | *TCAAGATCTAAAGACTGGCATCCACGATA* | *In gtrIc gene* | *Bgl*II |
| *GtrIVLp8NruIF* | *TCATCGCGAAAAATAACAGCTATCACTCC* | *In gtrIV gene* | *Nru*I |
| *GtrIVLp8NruIR* | *TCATCGCGATTTTTTGAACTTCATTCAAAAAAAC* | *In gtrIV gene* | *Nru*I |
| *GtrIVBodySmaIF* | *TCACCCGGGGACTCTGCTCTATCAACTGTG* | *In gtrIV gene* | *Sma*I |
| *GtrIVBodySmaIR* | *TCACCCGGGGCTTGGCGAGAAAAATGAAAATG* | *In gtrIV gene* | *Sma*I |
| *GtrIcLp10SmaIF* | *TCACCCGGGGTACCTAAAAAAAGTAGCCAC* | *In gtrIc gene* | *Sma*I |
| *GtrIcLp10SmaIR* | *TCACCCGGGCCTGTTTGAACCTATAAGTTG* | *In gtrIc gene* | *Sma*I |
| *GtrIcBodySmaIF* | *TCACCCGGGAGTAATCGCACTTGTCATCAAAC* | *In gtrIc gene* | *Sma*I |
| *GtrIcBodySmaIR* | *TCACCCGGGGTCGTATTAATATCTTTCATAGTA* | *In gtrIc gene* | *Sma*I |
| *Sequencing* | | | |
| *PhoSeqNewR* | *GCACCTTCGGCATAATTACGTGC* | *173 bp into phoA/lacZ coding sequence* |  |
| *M13R* | *GGAAACAGCTATGACCATG* | *pBS KS multiple cloning site* |  |

Primers have been grouped into categories that correspond to their main use. Some primers were used in multiple studies. Restriction enzyme sites on the primers are underlined. For primers used in mutagenesis, the bases that have been changed are in bold italics.

**Table S2:** Results from the computer programs used to predict GtrIV topology and the basis for each prediction.

| **Program** | **Basis for prediction** | **Reference** | **N-terminus** | **Number of predicted transmembrane helices** |
| --- | --- | --- | --- | --- |
| HMMTOP | Hidden Markov Model | Tusnady and Simon (1998) | In | 8 |
| SOSUI | Hydropathy profile | Hirkawa*et al*. (1998) | - | 10 |
| TMPred | Comparisons with database of known transmembrane proteins | Hofmann and Stoffel (1993) | In | 8 |
| DAS | Dense Alignment Surface Method | Czerzo*et al*. (1997) | - | 11 |
| TopPred | ‘Positive inside’ rule | von Heijne (1992) | Out | 9 |
| TMHMM | Hidden Markov Model | Sonnhammer*et al*. (1998) | - | 9 |
