# Supplementary figures and images for: Topological characterisation and identification of critical domains within glucosyltransferase IV (GtrIV) of Shigella flexneri
Source: BMC Biochem. 2011 Dec 22;12:67. doi: 10.1186/1471-2091-12-67 (PMC3259042; doi:10.1186/1471-2091-12-67)

## Slide 1
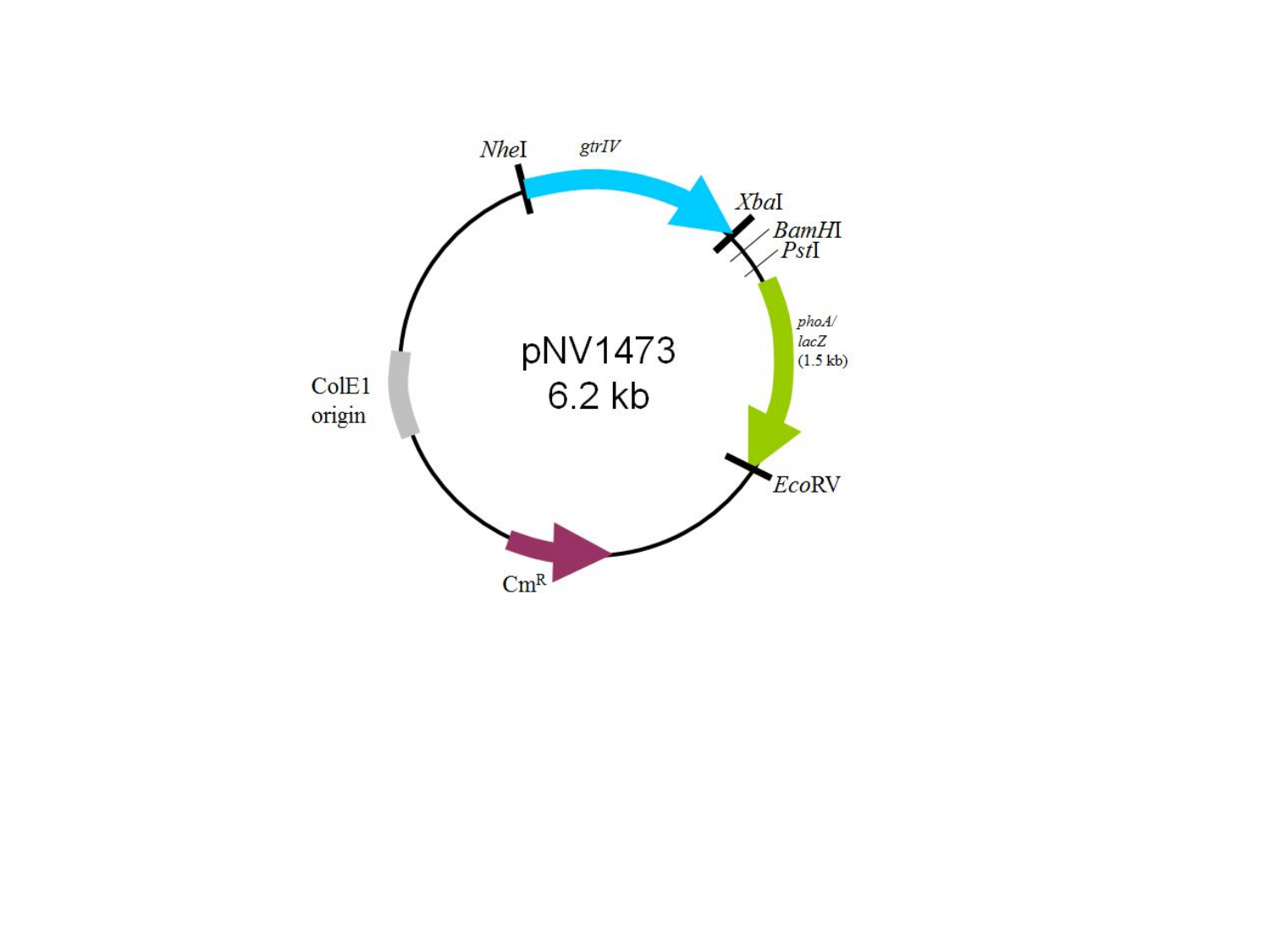

Supplement: Additional file 2 — Figure S1. The structure of pNV1473 depicting gtrIV and phoA/lacZ in tandem. [file 1471-2091-12-67-S2.PPT]

## Slide 1
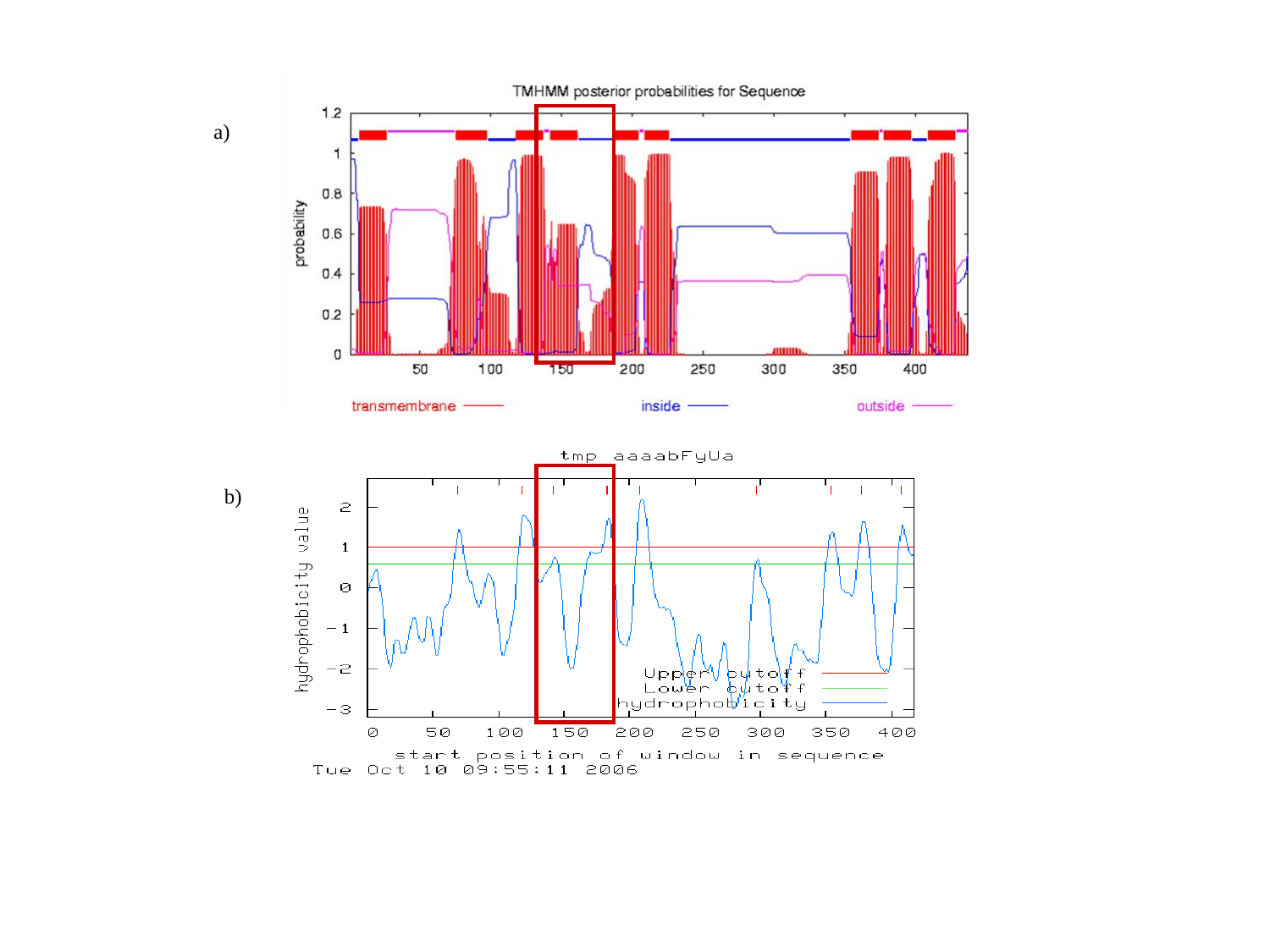

a)
b)
Supplementary Figure 2

Supplement: Additional file 4 — Figure S3. Representative computer-based topology predictions of GtrIV. [file 1471-2091-12-67-S4.PPT]
