# Supplementary material for: Topological characterisation and identification of critical domains within glucosyltransferase IV (GtrIV) of Shigella flexneri
Source: BMC Biochem. 2011 Dec 22;12:67. doi: 10.1186/1471-2091-12-67 (PMC3259042; doi:10.1186/1471-2091-12-67)
Supplement: Additional file 3 — Figure S2. The consensus topology of GtrIV. [file 1471-2091-12-67-S3.PPT]

## Slide 1
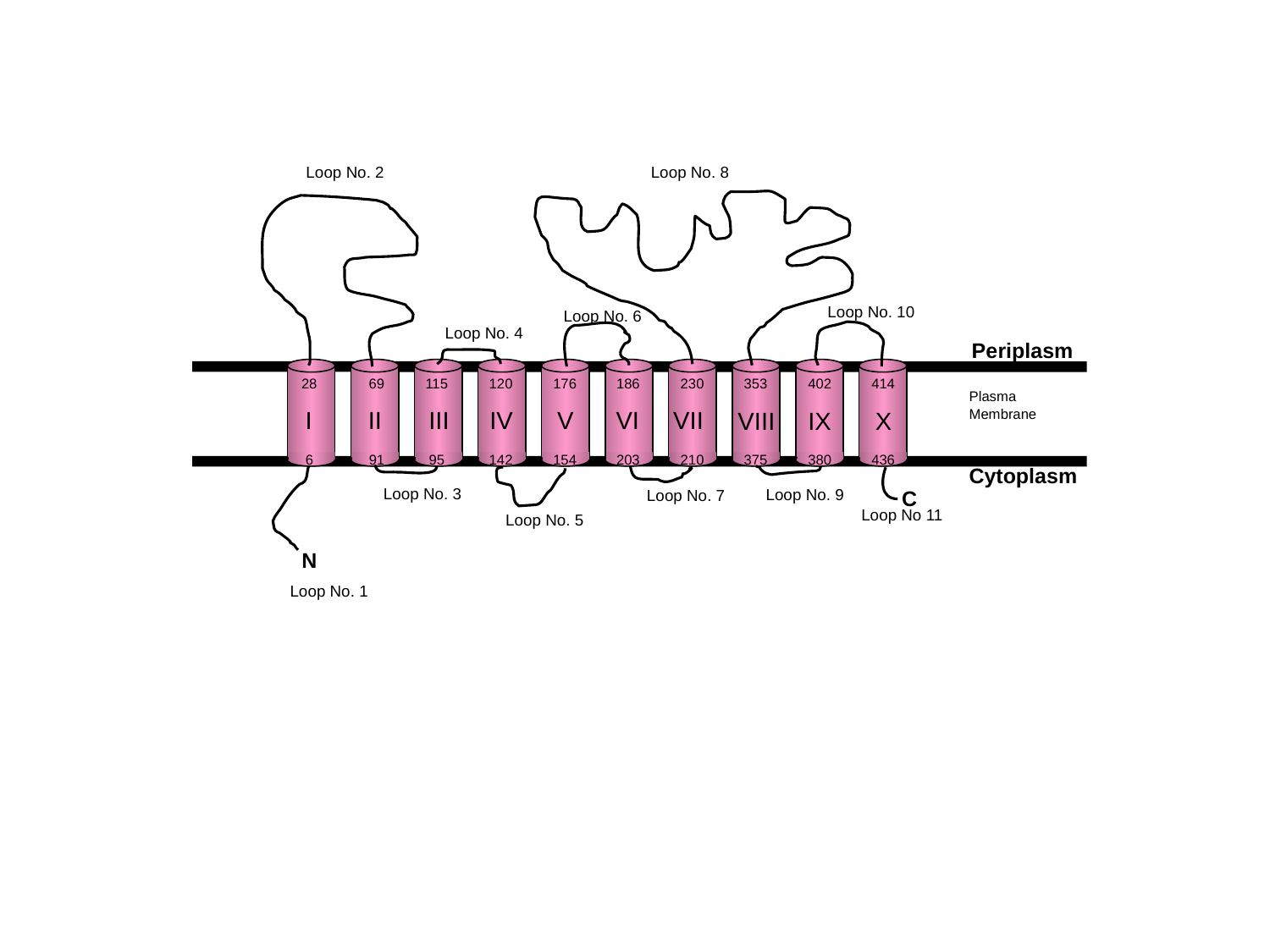

Loop No. 2
Loop No. 8
Loop No. 10
Loop No. 6
Loop No. 4
Periplasm
28
69
 115
120
 176
186
 230
353
 402
414
Plasma
Membrane
I
 II
 III
 IV
 V
 VI
 VII
 VIII
 IX
 X
 6
91
95
142
 154
203
 210
375
 380
436
Cytoplasm
Loop No. 3
Loop No. 9
C
Loop No. 7
Loop No 11
Loop No. 5
N
Loop No. 1
